# Mapping queen snapper (*Etelis oculatus*) suitable habitat in Puerto Rico using ensemble species distribution modeling

**DOI:** 10.1371/journal.pone.0298755

**Published:** 2024-02-26

**Authors:** Katherine E. Overly, Vincent Lecours

**Affiliations:** 1 Technical and Engineering Support Alliance, National Oceanic and Atmospheric Administration, National Marine Fisheries Service, Southeast Fisheries Science Center, Panama City, Florida, United States of America; 2 School of Forest, Fisheries, and Geomatics Sciences, University of Florida, Gainesville, Florida, United States of America; 3 Laboratoire d’expertise et de recherche en géographie appliquée, Université du Québec à Chicoutimi, Chicoutimi, Québec, Canada; MARE – Marine and Environmental Sciences Centre, PORTUGAL

## Abstract

Queen snapper (*Etelis oculatus*) is of interest from an ecological and management perspective as it is the second most landed finfish species (by total pounds) as determined by Puerto Rico commercial landings (2010–2019). As fishing activities progressively expand into deeper waters, it is critical to gather data on deep-sea fish populations to identify essential fish habitats (EFH). In the U.S. Caribbean, the critically data-deficient nature of this species has made this challenging. We investigated the use of ensemble species distribution modeling (ESDM) to predict queen snapper distribution along the coast of Puerto Rico. Using occurrence data and terrain attributes derived from bathymetric datasets at different resolutions, we developed species distribution models unique to each sampling region (west, northeast, and southeast Puerto Rico) using seven different algorithms. Then, we developed ESDM models to analyze fish distribution using the highest-performing algorithms for each region. Model performance was evaluated for each ensemble model, with all depicting ‘excellent’ predictive capability (AUC > 0.8). Additionally, all ensemble models depicted ‘substantial agreement’ (Kappa > 0.7). We then used the models in combination with existing knowledge of the species’ range to produce binary maps of potential queen snapper distributions. Variable importance differed across spatial resolutions of 30 m (west region) and 8 m (northeast and southeast region); however, bathymetry was consistently one of the best predictors of queen snapper suitable habitat. Positive detections showed strong regional patterns localized around large bathymetric features, such as seamounts and ridges. Despite the data-deficient condition of queen snapper population dynamics, these models will help facilitate the analysis of their spatial distribution and habitat preferences at different spatial scales. Our results therefore provide a first step in designing long-term monitoring programs targeting queen snapper, and determining EFH and the general distribution of this species in Puerto Rico.

## Introduction

Knowledge of the spatial distributions of marine species is necessary for the development and implementation of management strategies for fisheries around the world. The shift towards ecosystem-based fisheries management (EBFM) has become more mainstream in recent years, focusing on habitat, ecosystem processes, and the sustainability of populations [1, 2]. This approach to fisheries management requires accurate ecological information on the spatial distribution of key species, and critical environmental variables that influence observed patterns of habitat use [3]. We note that the term “habitat” has been used in many different ways by marine scientists [4]; in this study, we are using the term “habitat” as the combination of environmental characteristics, and in particular characteristics of the physical environment, that is associated with the presence of a species at given spatial and temporal scales [4].

Identification, mapping and understanding Essential Fish Habitats (EFH) provide important spatial information to support EBFM. EFH has been broadly defined in the Magnuson-Stevens Fishery Conservation and Management Act as “waters and substrate necessary for fish spawning, breeding, feeding or growth to maturity,” making it difficult to discern what in fact is essential about EFH [5]. To begin to determine EFH for a species, basic distribution data must be available for a species’ geographic range. In the U.S. Caribbean, a lack of ecological information on deepwater fish species has made it difficult to define EFH for the suite of species occupying those domains. However, recent improvements in habitat mapping technologies and underwater video systems have greatly advanced our ability to generate spatially-explicit data, particularly for deepwater habitats [6, 7]. Within U.S. waters, studies on the distribution and species assemblages of Caribbean deepwater habitats are limited when compared to the Gulf of Mexico or other parts in the North Atlantic Ocean. Yet several species of deepwater snapper, including queen snapper (*Etelis oculatus*), have previously been determined to be undergoing overfishing, or their stock status is unknown within the U.S. Caribbean Exclusive Economic Zone [8]. The life history of queen snapper is characterized by slow growth and high longevity [9, 10], similar to that of other deepwater fishes [11, 12], thus rendering the species vulnerable to, and likely slow to recover from, fishing pressure. This is particularly critical for data-poor species for which management decisions will undoubtedly deal with uncertain model parameters, spatial distribution models, relative abundance indices, and diet matrices (in the case of EBFM). As such, identifying and understanding EFH is crucial for the long-term biological and economic sustainability of fisheries and deepwater habitats along Puerto Rico’s coast.

**Fig 1:**
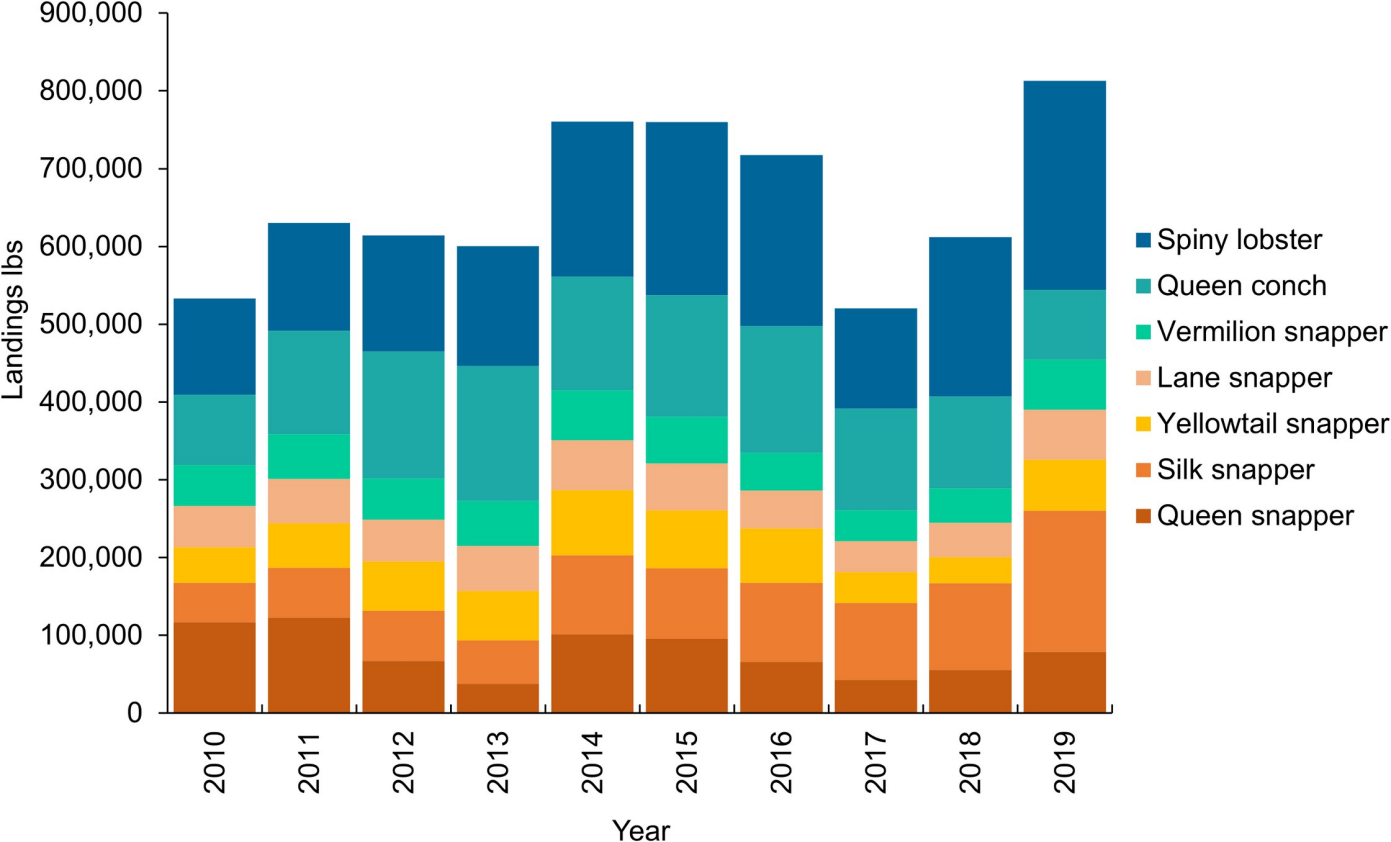
Deepwater finfish landings in Puerto Rico. Total Puerto Rico commercial landings, in pounds (lbs), by year for the top five reef-associated finfishes, spiny lobster, and queen conch.

Queen snapper is of interest from an ecological and management perspective as it is a targeted component of the commercially important deepwater snapper-grouper complex fishery found throughout Puerto Rico and the Caribbean. In Puerto Rico, queen snapper is the fourth most landed species, and the second most landed finfish (Fig 1) according to the National Oceanic and Atmospheric Administration (NOAA) Trip Interview Program data (2010–2019), and in recent years the fishing effort targeting this species appears to have shifted to deeper waters, possibly in response to stock depletion [13]. Yet, little is known of its fine-scale distribution patterns and the habitats it utilizes. A study conducted by Cummings [14] indicated that queen snapper is most abundant in areas characterized by rocky bottom habitat near oceanic islands, and Allen [15] noted their adult depth range of 130–450 m. However, researchers onboard the NOAA exploratory research vessel Okeanos Explorer recently established a new maximum depth for the species of 534 meters (m) with direct observations via a remotely operated vehicle [16]. Knowledge of juvenile habitat and depth range is more limited. A study conducted by Gobert *et al*. [17] noted several fish between 55–70 millimeter (mm) fork length (FL) were found as deep as 490 m, whereas the smallest fish obtained by Overly [10] was 178 mm FL, or 4 years old, captured at a depth greater than 100 m. Larvae appear to move deeper with ontogeny [18] and were found as deep as 100 m at 38 days old. Thus, it is difficult to discern if there are age-based differences in habitat use for queen snapper.

Effective management strategies for queen snapper in the U.S. Caribbean will require a knowledge of their spatial distribution to not only define EFH, but also guide future fishery surveys and identify exploited and unexploited regions. Consequently, the Caribbean Fishery Management Council (CFMC) has prioritized investigations into the deepwater snapper-grouper complex, particularly the habitats it targets [19]. However, as a relatively deepwater species, it is difficult to develop expansive occurrence datasets for queen snapper due to limited opportunities and costly field sampling. In addition, describing deepwater habitats is challenging mainly due to technological hurdles associated with visually assessing deepwater benthos.

Marine habitat mapping has become a critical first step in EBFM [20] and can combine environmental variables at sites of known species occurrence to predict a species’ distribution in unsampled areas and explore habitat suitability [6, 21]. In particular, species distribution models (SDM) enable the exploration of species-environment relationships that can help infer potential environmental or ecological requirements needed by a particular species. Presence-only, or presence-background SDMs have gained traction in recent years as presence/absence models are not necessarily well suited for marine species modeling [22, 23]. Monk *et al*. [21] indicate a bias towards falsely identifying absences in the marine environment due to the explicit constraints surrounding absence data, primarily what constitutes a true absence versus a failure to detect. The issue of false absence can be exacerbated when sampling cryptic species or taking into account the sampling bias (e.g., selectivity) of certain gear types [24]. Classifying sites where a fish was not seen or caught as non-suitable habitat has the potential for inaccuracy due to bias surrounding the nature of sampling and thus estimating error. Presence-background modeling allows for estimating spatial distributions of a species’ niche habitat from positive observations in the data. In addition, presence-background SDM is less sensitive to small sample sizes (n < 30) while still generating ecologically valuable models, which is of particular importance in the deep oceans where sampling and data are limited [21, 25–29].

In more recent years, ensemble species distribution modeling (ESDM) has been used for marine fishes and as an approach to marine benthic habitat mapping [27, 30]. Outcomes demonstrate that in addition to providing sounder results through measurements of uncertainty [30, 31], ESDMs also tended to outperform individual SDMs with increased precision measured by the area under the receiver operating characteristic curve (AUC) [29, 32], and true skill statistic (TSS) metrics [32]. The benefit of training multiple algorithms that differ in their predictions is twofold: the ensemble model is typically more accurate than any individual model on its own; and by combining models with varying structures, we can ensure diverse classification results that focus solely on multiple classifications simultaneously [33]. In addition, ESDM enables the quantification of model uncertainty, a valuable product when models are used to inform decision-making and management [34].

In this study, we used ESDM to predict areas of suitable habitat for queen snapper, and by extension map their potential distribution along sections of the coast of Puerto Rico. The objectives of this work were to: 1) develop robust habitat suitability and uncertainty maps for each of the study regions; and 2) quantify species-environment relationships to evaluate the potential of various environmental variables, and more specifically terrain variables, to act as surrogates for queen snapper distribution. Our main goal through modeling is exploratory in nature as we sought to identify areas where queen snapper habitat suitability is predicted to be high (≥ 85%) and identify the available variables explaining queen snapper distribution the most. The models developed in this study are intended to be used as a tool to identify potential areas in which queen snapper may be found, in a more cost-effective way than intensive biological sampling. Analyzing habitat utilization and the distribution of queen snapper will not only add to our limited knowledge regarding queen snapper habitat preferences, but results could also be incorporated into spatial planning under EBFM and the start of determining EFH for queen snapper.

## Materials and methods

### Species occurrence data

**Fig 2:**
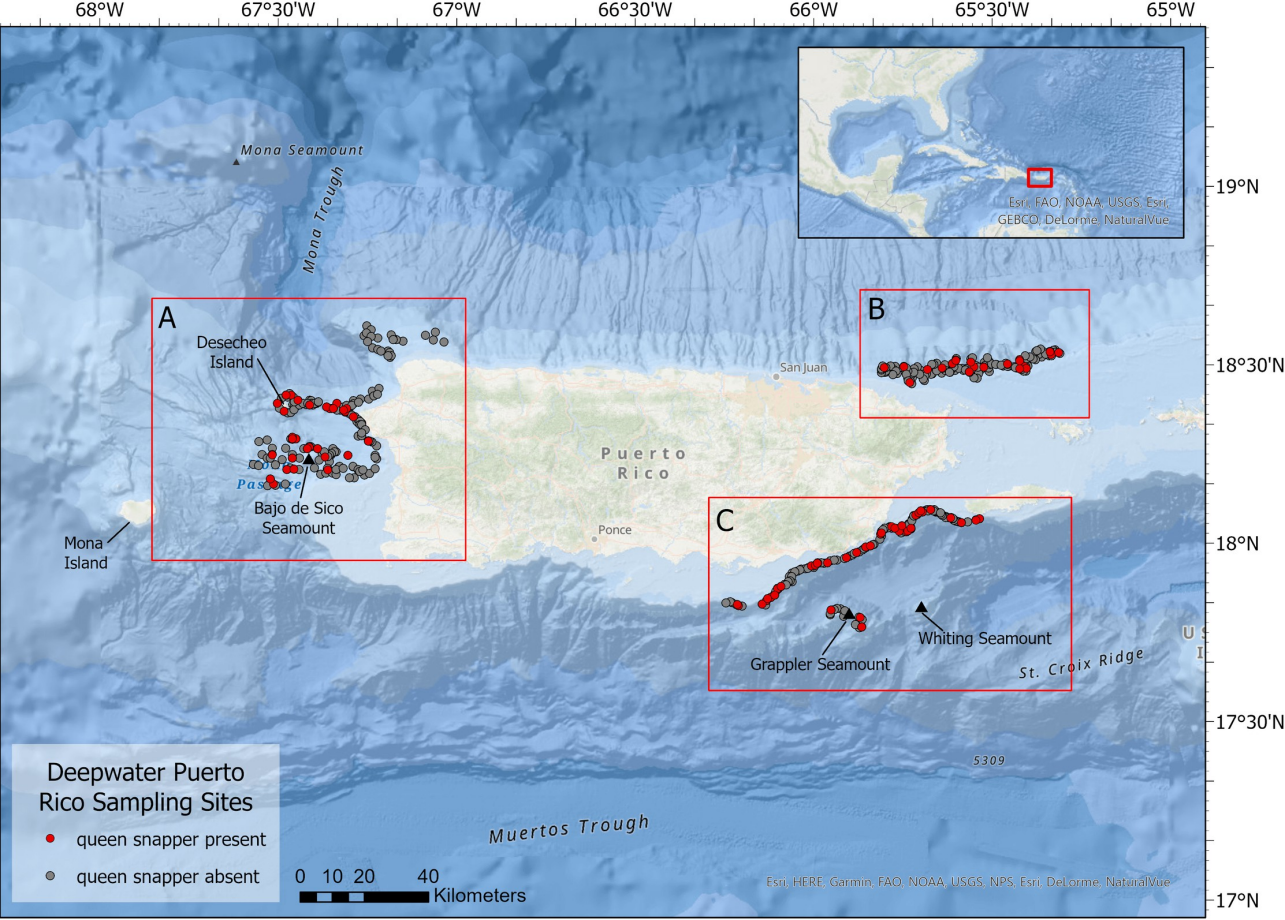
Deepwater Puerto Rico sampling sites. Map of all survey sites sampled in two years with a remote video camera and hook and line fishing along the western (A), northeastern (B), and southeastern (C) coasts of Puerto Rico. The map layer used to generate this figure is from the NOAA National Centers for Environmental Information and provided without restriction by the U.S. Government.

Queen snapper occurrence data were collected during a fishery-independent, video and hook and line survey, conducted between 2018 and 2020 in depths ranging from 100–500 m (Fig 2). Sites were selected using a stratified random sampling design that utilizes a combination of depth gradient and habitat, with sites allocated by 50 m depth intervals along three classes of habitat complexity: low, moderate, and high as defined by an ArcCord rugosity score developed by the University of Miami Rosenstiel School of Marine, Atmospheric, and Earth Science [35].

**Fig 3:**
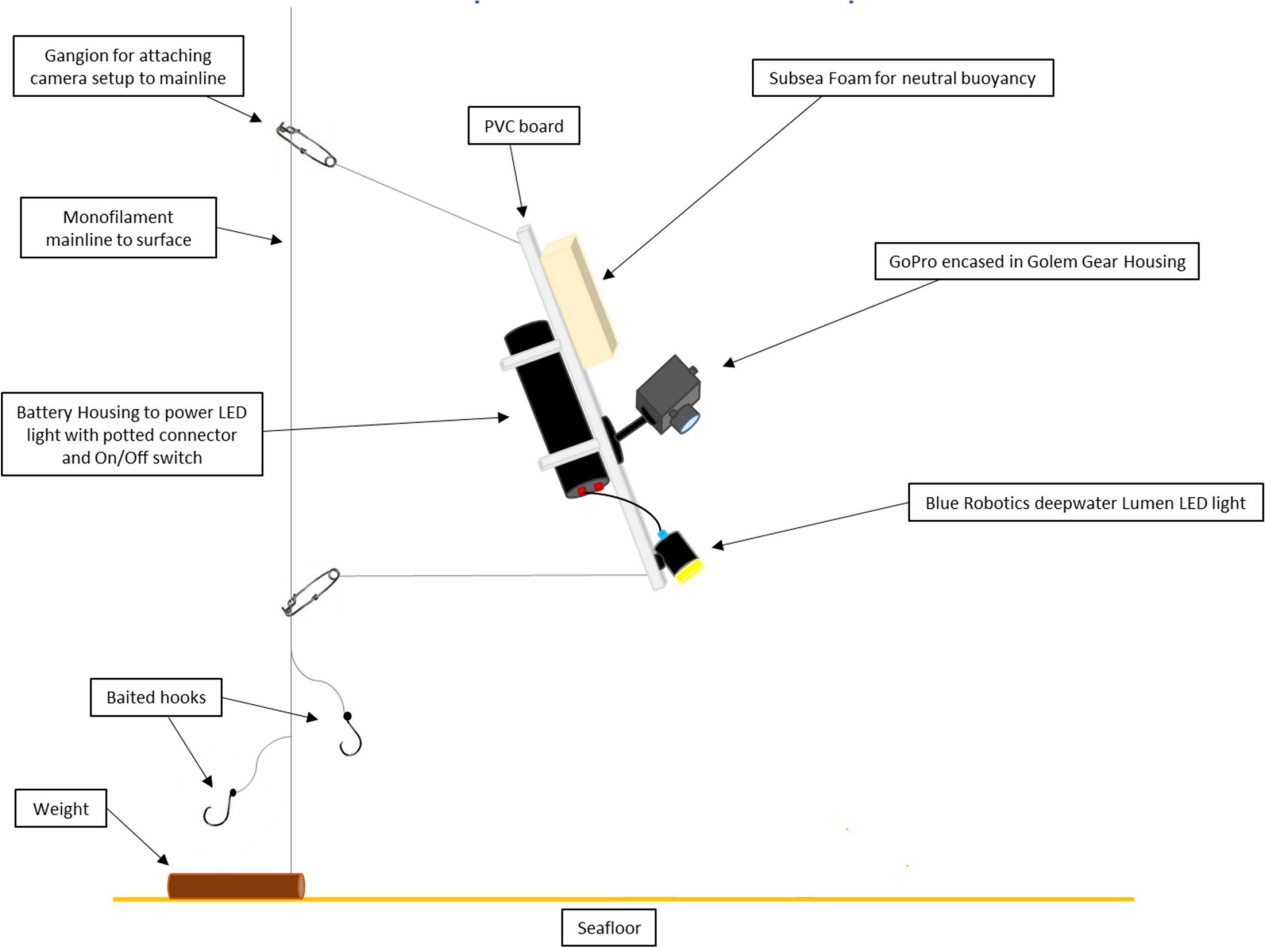
Deepwater video camera system diagram. Deepwater video camera system, including a GoPro camera and Golem Gear housing with an attached LED light and battery housing. Subsea buoyancy foam allows the system to achieve neutral buoyancy, oriented at 45 degrees to the seafloor. The camera system is attached to the fisher’s vertical hook and line with two gangions. Below the camera system are two baited hooks, and a weight to keep the line stationary. Reprinted under a CC BY license, with permission from Katherine Overly, original copyright 2019.

The project utilized Puerto Rican commercial fishers to conduct camera and fishing gear deployments. Each deployment consisted of two separate vertical lines at each selected site’s coordinates, the first targeting queen snapper via hook and line fishing and the second the associated habitat and species presence/absence via a video camera system. Each vertical line was composed of monofilament and synthetic braided line rigged with a 4.5-kilogram (kg) weight attached to the bottom of the line. The first line deployment incorporated 12 leaders with 9/0 Mustad Extra Wide® circle hooks baited with California squid (*Loligo sp*.). The fishing line was deployed for a total bottom time of 15 minutes after the weight reached the seafloor. The second line, deployed at the same coordinates as the hook and line, consisted of two baited hooks and the camera system. The camera system was deployed for a total bottom time of five minutes after the weight hit the seafloor and was then retrieved. To sample the full range of depths, a customized video camera system had to be created that not only allowed sampling to a depth of 500 m, but also provided lighting as light penetration at mesophotic-deep benthic reefs is limited. The video camera system consisted of a Golem Gear® housing enclosing a GoPro HERO3® high-definition camera, deepwater LED lights from Blue Robotics® and Sartek® Industries, and an aluminum battery housing enclosing a lithium-ion battery (Fig 3). The system consists of several pieces of white marine-grade high-density polyethylene sheets constructed in such a way as to reduce drag upon deployment and retrieval. Two pieces of syntactic foam coated in epoxy were attached to the camera system to attain neutral buoyancy with the GoPro’s field of view angled towards the seafloor. The camera rig was tethered to a vertical fishing line with two gangions approximately one meter above two 9/0 Mustad Extra Strong® circle hooks and a bottom weight.

### Environmental data

**Fig 4:**
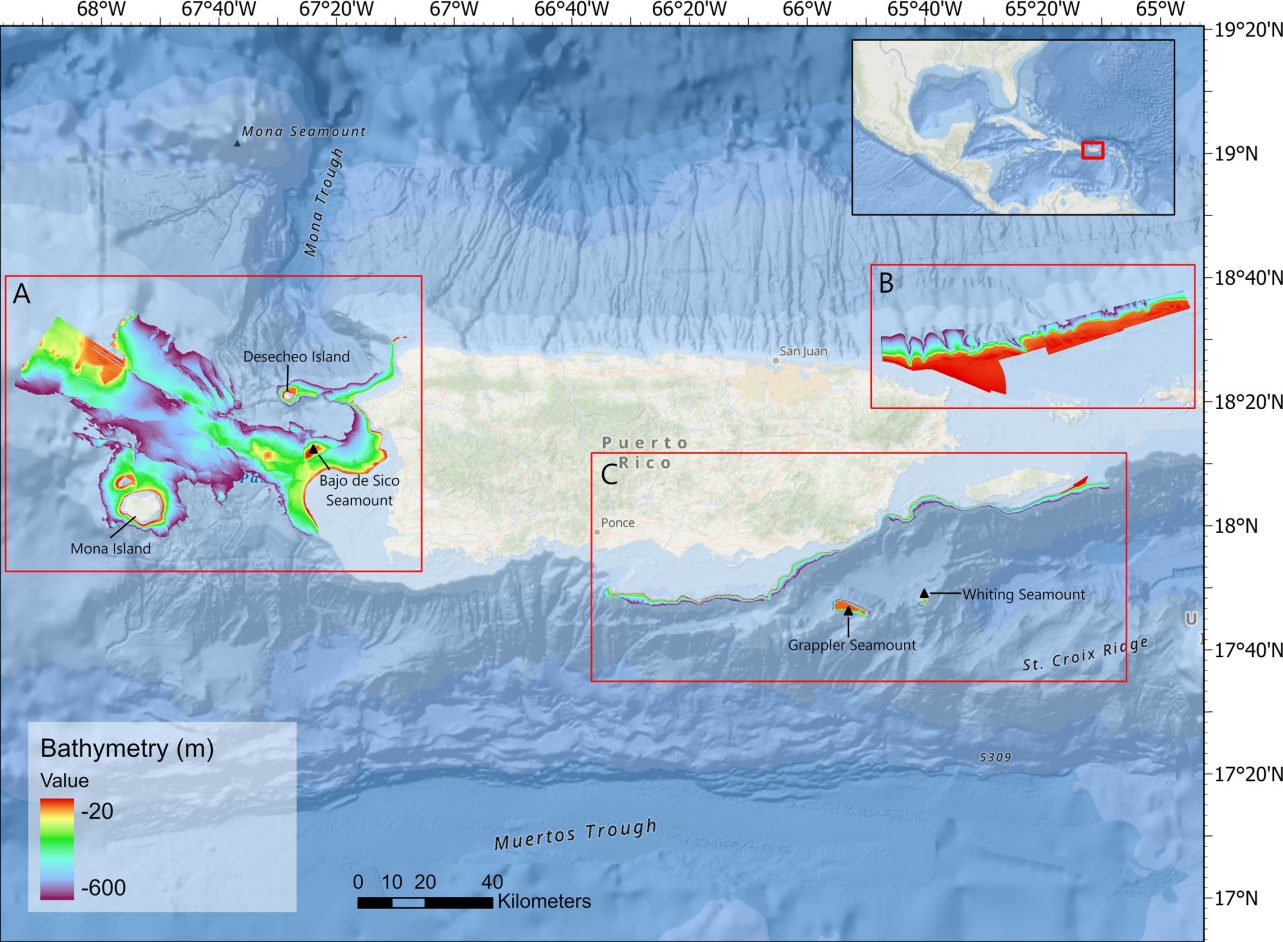
Bathymetric map of Puerto Rico. Map depicting mosaicked bathymetric data used in this study for the west (A), northeast (B), and southeast (C) regions of Puerto Rico. All depths are in meters (m). The map layers used to generate this figure are from NOAA National Centers for Environmental Information and provided without restriction by the U.S. Government.

The targeted study area was limited by the availability of bathymetric data provided by NOAA’s National Centers for Coastal Ocean Science (NCCOS) and encompassed three major fishing regions off the coast of Puerto Rico: the west, northeast and southeast (Fig 4). Available bathymetric data were collected in prioritized regions by NOAA NCCOS and the United States Geological Society using multibeam echosounder systems (MBES). Due to limited mapping data and differing resolutions between regions (2 m, 4 m, 8 m, and 30 m), multibeam bathymetry rasters were mosaicked in ArcGIS Pro (v10.1) to a spatial resolution of 8 m on the northeast and southeast coasts, and to 30 m on the west coast (Fig 4). These two resolutions showed the best compromise to explore the effect of differing spatial resolutions on capturing queen snapper habitat preference and to produce the highest possible resolution multibeam data, which is lacking in many regions in the U.S. Caribbean, particularly on the west coast.

As commonly performed in habitat mapping studies [4, 36], multiple terrain attributes were derived from the bathymetry data collected at each location. The terrain attributes were selected based on several studies that determined an optimal selection of variables for species distribution modeling in the marine environment [3, 34, 37]. The ArcGIS Pro Spatial Analyst extension was used to derive the slope, slope of slope, general curvature, planform curvature, and profile curvature. The Benthic Terrain Modeler (BTM) toolbox in ArcGIS was used to compute the vector ruggedness measure (VRM), fine-scale benthic position index (BPI) using and inner radius of 5 and an outer radius of 25, and broad-scale BPI using an inner radius of 25 and an outer radius of 250 [38]. The TASSE toolbox was used to derive relative distance from mean value (*i*.*e*., a measure of relative position), standard deviation (*i*.*e*., a measure of rugosity), and northerness and easterness (*i*.*e*., non-circular derivatives of aspect, the orientation of the slope) [34, 36, 38]. Given the availability of bathymetric data in the sampling universe (Fig 4), depth was limited to the 0 to 600 m range, which encompassed the queen snappers’ observed depth range and slightly beyond. By delimiting depth, the potential for there to be a misleading effect on model performance is reduced through the reduction of large sections of depths with known zeros where pseudoabsence data would typically be derived [39]. Correlation analyses were performed by way of a correlation matrix in R using the ‘corrplot’ package to reduce the likelihood of model overfitting, uncorrelated variables (Spearman’s correlation coefficient ∈ [-0.65, 0.65]) were retained for modeling.

### Modeling

Individual ESDMs were developed and run using the statistical software ‘R’ and the package ‘SSDM’ in each region [40, 41]. Algorithms in the SSDM package that were run included: generalized boosted regression models (GBM), multivariate adaptive regression splines (MARS), classification tree analysis (CTA), random forest (RF), maximum entropy (MAXENT), artificial neural network (ANN) and support vector machine (SVM). Because the presence data in the northeast (n = 20) was slightly less than in the west (n = 47) and southeast (n = 42), the algorithms for generalized additive models (GAM) and generalized linear model (GLM) were not included in the ensemble modeling due to more stringent sample size requirements. The models were supplied with occurrence records and calibrated to pick pseudo-absence points using the default strategy [42] incorporated within the SSDM package. The default strategy included: 1) the averaging of several runs with fewer pseudo-absences with equal weighting for presences and absences for MARS and discriminant analyses; 2) the use of the same number of pseudo-absences as available presences for techniques such as GBM, CTA, and RF; 3) the random selection of pseudo-absences when using regression techniques; and 4) the random selection of geographically and environmentally stratified pseudo-absences when utilizing classification and machine-learning techniques [42]. To reduce the likelihood of spatial autocorrelation, geographic resampling of data was incorporated into the model runs using the R package for spatial thinning of species occurrences “spThin” [43]. This R package uses a randomization approach to thin occurrence datasets, creating new subsets that meet a minimum nearest neighbor distance constraint of two pixels. Individual SDMs were trained and tested using the default parameters of the dependent R package of each statistical method (S1 Table). The highest-performing algorithms (as determined by Cohen’s Kappa coefficient; Kappa ≥ 0.70) for each region were retained in the ESDM. To ensure independence between the training and evaluation sets for cross-validation and to combat issues with the metrics influence on the selection of models, the “holdout” method was implemented in the modeling workflow with ten iterations [41]. This method allowed for a subset of data independent from the models to be used in the evaluation, e.g. a separate training and evaluation set, leaving out 30% of the presence records and pseudo-absences randomly, calibrating with 70%, and then measuring the model performance with the independent points in each model.

The ESDM was created for each region from the highest performing SDM’s, capturing components from each. To form a consensus among the highest performing SDM projections, a simple average of the model outputs was taken [41], resulting in a consensus ESDM for each region. ESDMs were verified using a ten-fold cross-validation procedure. The ESDMs generated a measure of uncertainty (between-methods variance), which was calculated for each ensemble model, in addition to the AUC, sensitivity, specificity, omission rate, proportion of correct predictions, Cohen’s Kappa coefficient.

There has been some debate on whether the AUC adequately assesses the accuracy of the predictive distribution models, despite its tendency to be reported as a single measure of overall model performance [44–49]. The main cause for concern is that the AUC/ROC curve itself does not reflect the true performance of the model [48], although it does provide information regarding the degree to which a species is restricted to any particular part of a range of predictor variables, i.e., presence/absence. AUC/ROC plots specifically require true absences [50] to calculate the AUC metric. Identifying true absences is a very complex issue in a marine environment and is made more difficult by species such as the queen snapper, which is mobile. As a result, we cannot say for certain that areas where queen snapper were not caught or observed on video are true absences. To address accuracy concerns, additional metrics were utilized that take into account the importance of both commission and omission errors within the models. The ESDM’s performance was additionally evaluated with the partial AUC/ROC (pAUC/ROC), which leaves out the evaluation of absences and concentrates specifically on the evaluation of presences [51]. For the pAUC/ROC, the proportion of error allowed was set to 0.05, and 500 iterations were used for the bootstrap. Variable relative importance was evaluated based on a jackknife approach between a full model and a model with each environmental variable omitted in turn [52]. The Pearson metric was utilized, which computed a simple Pearson’s correlation (r) between predictions of a full model and one omitting a variable, computed as 1- r. The higher the return value, the more influence the variable has on the model. To reduce the risk of model over-fitting, variables with a variable relative importance of ≤ 3.0% were removed and the ESDM was re-run without the omitted variables.

Binary maps showing suitable and unsuitable locations were generated using the SSDM package. The optimal threshold to split presences and absences on the basis of habitat suitability probabilities was first set to the probability that maximizes the TSS, or the sum of the sensitivity and specificity [52, 53]. The results using the default TSS were evaluated and compared to what we currently know of the species biology and ecology. Consequently, the models were rerun using differing thresholds as were seen fit and re-evaluated using the standard protocol for reporting SDMs called Overview, Data, Model, Assessment and Prediction (ODMAP) [44].

## Results

A total of 471 sites were sampled over the course of the two-year project contributing queen snapper presence data (Fig 2). Because queen snapper individuals appear to shy away from the white LED light wavelength, and the bottom time for the video camera system was short, they were not commonly seen on video over the two-year survey. Consequently, sites where queen snappers were caught using hook and line methods were added to the sites where queen snappers were positively identified on video to be utilized in the modeling (n = 109; west n = 47, northeast n = 20, southeast n = 42). All occurrences were retained following the spatial thinning process.

### Variable selection

Of the 13 derived terrain attributes, bathymetry, slope, VRM, and fine-scale BPI, were retained in the final models in all three regions (correlation coefficient < |0.65|; Table 1). Additionally, broad-scale BPI and profile curvature were retained in the northeast and west, respectively, and northerness was retained in the northeast and southeast. Curvature, standard deviation, RDMV, and slope of slope were found to be highly correlated in all three regions and were not included in the ESDMs; likewise, broad-scale BPI was found to be highly correlated in the west and southeast and removed from analysis (correlation coefficient of > |0.65|; Table 1). Plan curvature and easterness contributed minimally to variable relative importance (< 3.0%) in the three regions and were removed from the ensemble modeling; likewise, profile curvature in the northeast and southeast and northerness in the west were found to contribute minimally to variable relative importance and were removed from modeling (Table 1).

**Table 1:** Summary of variables retained for final ensemble species distribution models.

Variable	Region retained after correlation analysis^a^	Region retained after variable importance analysis^b^
*Bathymetry*	West, Northeast, Southeast	West, Northeast, Southeast
*Slope*	West, Northeast, Southeast	West, Northeast, Southeast
*Slope of Slope*	None	None
*Curvature*	None	None
*Plan Curvature*	West, Northeast, Southeast	None
*Profile Curvature*	West, Northeast, Southeast	West
*Vector Ruggedness Measurement (VRM)*	West, Northeast, Southeast	West, Northeast, Southeast
*Fine-scale BPI*	West, Northeast, Southeast	West, Northeast, Southeast
*Broad-scale BPI*	Northeast	Northeast
*Standard Deviation*	None	None
*Relative Deviation from Mean Value (RDMV)*	None	None
*Easterness*	West, Northeast, Southeast	None
*Northerness*	West, Northeast, Southeast	Northeast, Southeast

^a^Environmental variables derived from bathymetric data and retained for modeling in ESDM after correlation analysis. Variables were not retained if Spearman’s coefficient was > |0.65|.

^b^Variables retained following interpretation of variable relative importance. Variables with relative importance ≤ 3.0% were removed from models.

### Comparison of individual algorithm models

The Cohen’s Kappa coefficient, or Kappa value, which measures the extent to which the agreement between observed and predicted values is higher than what would be expected by chance alone, was used to select the highest performing algorithms for use in producing the ESDM’s (Kappa value > 0.70). Kappa values for the retained algorithms provided model predictions ranging from almost perfect agreement (*i*.*e*., Kappa between 0.81 and 1.00) to substantial agreement (*i*.*e*., Kappa between 0.61 and 0.80; Table 2) [45], depending on region (Table 2). In the western region, GBM, RF and SVM algorithms resulted in Kappa values > 0.70, or substantial agreement [45], and were retained for use in the ESDM (Kappa values = 0.71, 0.71 and 0.78, respectively). The northeast region results were more varied, with the five algorithms obtaining Kappa values > 0.70, including ANN, CTA, GBM, RF and SVM (Kappa values = 0.75, 0.83, 0.77, 0.90, and 0.86, respectively; Table 2) ranging from substantial to almost perfect agreement [45]. The highest performing algorithms selected in the southeast region were GBM, RF, and SVM (Kappa values = 0.76, 0.76, and 0.80, respectively; Table 2).

**Table 2:** Species distribution models retained in the ensemble species distribution models.

Region	MAXENT	RF	MARS	GBM	CTA	ANN	SVM
West	-	0.71	-	0.71	-	-	0.79
Northeast	-	0.90	-	0.77	0.83	0.75	0.86
Southeast	-	0.76	-	0.76	-	-	0.80

Cohen’s Kappa Coefficient values for the retained SDMs for the three regions of Puerto Rico. NR = Kappa value < 0.70

### Ensemble species distribution model

**Fig 5:**
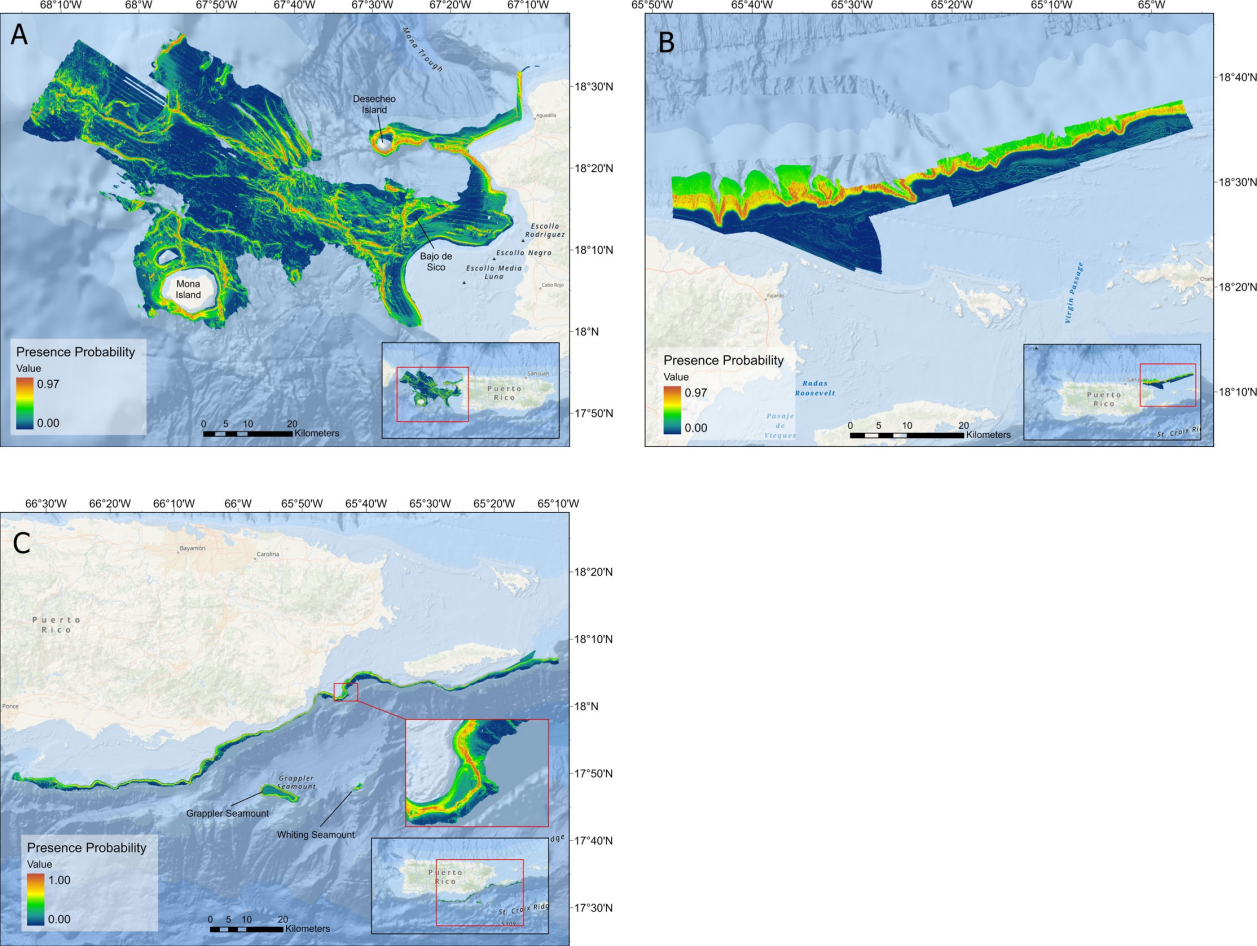
Region-specific presence probability maps. Queen snapper habitat suitability maps for the A) west, B) northeast, and C) southeast region of Puerto Rico. The map layer used to generate this figure is from NOAA National Centers for Environmental Information and provided without restriction by the U.S. Government.

An ESDM was generated for each study region to analyze fish distribution and habitat suitability at different spatial resolutions using the highest performing approaches for each region: west—GBM, RF, and SVM; northeast–ANN, CTA, GBM, RF, and SVM; and southeast—GBM, RF, SVM. The predictability of queen snapper presence was generally high across the three ESDMs, with suitable habitat presence probabilities exceeding > 95% in areas of all three regions (Table 3 and Fig 5). The generated suitable habitat presence probability maps show that on the west coast, queen snapper presence aligns with the presence of larger bathymetric features. For example, the probability of occurrence around the seamounts Bajo de Sico, Mona Island, Desecheo Island, and large ridge features throughout the Mona Passage reach up to 97%. On the northeast coast, queen snapper suitable habitat presence probability appeared to be highest around the canyon-like features with some areas displaying probabilities as high as 97%. Suitable habitat presence probability in the southeast somewhat mirrored the west, with the highest presence probability of 100% localized to areas near the extremely steep continental slope and neighboring seamounts, Grappler, and Whiting.

**Table 3:** Results for region-specific ensemble species distribution models.

Region	Threshold	AUC	Omission Rate	Sensitivity	Specificity	Proportion Correct	Kappa
West	0.32	0.87	0.14	0.86	0.88	0.87	0.74
Southeast	0.38	0.89	0.11	0.94	0.82	0.89	0.77
Northeast	0.66	0.92	0.09	0.97	0.86	0.91	0.82

Results of Ensemble Species Distribution Modeling for each study region in Puerto Rico.

Overall, we found the range in AUC values, coupled with the sensitivity and specificity metrics, and Cohen’s Kappa coefficient, provided evidence that the ESDM’s had excellent predictive capabilities. AUC evaluation was conducted using the metric interpretations of Hosmer and Lemeshow [46] where an AUC value equal to 0.5 is interpreted as ‘no discrimination’, 0.5 < AUC ≤ 0.7 as ‘poor’, 0.7 < AUC ≤ 0.8 as ‘acceptable’, 0.8 < AUC ≤ 0.9 as ‘excellent’, and an AUC > 0.9 as ‘outstanding’. The ensemble models for the three regions in Puerto Rico provided ‘excellent’ to ‘outstanding’ (west, AUC = 0.87; northeast, AUC = 0.92; southeast, AUC = 0.89) predictive capability [46] and highlight the models’ ability to correctly rank occurrences above background locations. The sensitivity metric, which is the proportion of true positives, or fish that are both predicted and observed to be present, was high for all ESDMs (0.86–0.97) (Table 3). In addition to the sensitivity metric, the specificity metric, which is the proportion of true negatives, or the fish that are both predicted and observed to be absent, was high for all ESDMs (0.82–0.88) (Table 3). The true positive rate tended to outperform the true negative rate in all three regions, showing a slight tendency for the model to misclassify fish absence between the predicted and observed. The Kappa value refers to how representative the data collected are to the variables that were measured. The value can be interpreted as Kappa ≤ 0 is ‘no agreement’, 0 > Kappa ≤ 0.2 is ‘slight’, 0.2 > Kappa ≤ 0.4 is ‘fair’, 0.4 > Kappa ≤ 0.6 is ‘moderate’, 0.6 > Kappa ≤ 0.8 is ‘substantial’, and 0.8 > Kappa ≤ 1.0 is ‘almost perfect’ [45]. The Kappa value for all three regions was > 0.70, indicating ‘substantial’ to ‘almost perfect’ model accuracy [45]. While these metrics indicate that the models performed well, they are not necessarily rigorous indicators of model performance due to issues with delineating true absence as described above [51]. By implementing the holdout method, we were able to leave out the evaluation of absences entirely, concentrating solely on the evaluation of presences. This evaluation metric enabled us to accept an omission error level and test via hypothesis (Ho: pAUC ≤ 0.5) [51]. The mean value for the pAUC/ROC at random for all final ESDMs was < 0.50, indicating that our resulting models are better than chance alone.

### Binary maps

Probabilities of suitable habitat were converted to a binary index of habitat suitability for the three regions. An optimal threshold was first determined by the default parameters in the SSDM package, as determined by maximizing the sum of the sensitivity and specificity metric (Table 3), and used to convert the habitat suitability map into binary presence (*i*.*e*., a value of one) and absence (*i*.*e*., a value of zero) maps. For the first iteration run, the TSS metric was used to determine the threshold of probability separating probable presence from probable absence. This threshold varied per ESDM from 32% in the west, 66% in the northeast, and 38% in the southeast. Using the aforementioned thresholds, critical evaluation of the preliminary results determined that they did not meet what we know of the species distribution from field observations concerning bathymetry, meaning that predicted probable presence were located in areas where true absence are known. Using the ODMAP protocol, varying thresholds were explored and were manually adjusted to 70%, 80%, 85%, and 90% in the three regions (Tables 4 and 5) [44]. After critical evaluation, the thresholds of 85% and 90% were determined to be the most representative of the species known range, and 85% was used to determine the final habitat suitability maps. Our results suggest that care should be taken when determining thresholds in ESDM, and highlight the difference between what statistical tools provide, versus the critical evaluation of resulting maps and models. While the performance metrics of the ESDMs in each region were high, model performance is different from ecological realism. Queen snapper are known to inhabit depths of 100–534 m, with our models predicting > 85% suitable habitat presence probability that queen snapper resided in depths ranging from 160–429 m island-wide in Puerto Rico [10]. It is critically important that modelers also consider the underpinning ecology, such as ground-truthing presence observations to QA/QC model predictions to ensure the models are representative of the reality for the species. In our case, this was accomplished by manually setting the threshold to explore the variables’ mean and range. In critically evaluating our resulting models, we can determine that while spatial modeling tools provided in GIS or statistical packages have many easy-to-use tools, they may not provide results that are completely representative of what we know of the species biology and ecology.

**Table 4:** Region-specific ranges for the retained variables within suitable habitat.

Region	Threshold	Model Prediction					
**West**		***Slope***	***Bathymetry (m)***	***VRM***	***Fine-scale BPI***	***Profile Curvature***	
	32%	0.02–67.60	20–600	0.00000012–0.230	-208–188	-11.2–14.1	
	70%	5.00–67.60	21–459	0.00000520–0.230	-111–188	-11.2–12.9	
	80%	8.80–66.90	21–436	0.00015400–0.170	-85–188	-9.50–3.50	
	85%	9.00–54.70	187–429	0.00025600–0.069	-4–129	-4.10–1.45	
	90%	10.10–39.30	225–410	0.00067000–0.015	4–98	-1.64–0.76	
**Northeast**		***Bathymetry (m)***	***Broad-scale BPI***	***Northerness***	***Slope***	***Fine-scale BPI***	***VRM***
	66%	159–599	-244–161	-1- 1	0–81.7	-244–161	0–0.203
	70%	160–592	-211–141	-1- 1	1.3–80.7	-211–141	0.000099–0.182
	80%	160–400	-210–91	-1- 1	1.3–61.8	-210–91	0.000099–0.142
	85%	160–390	-200–90	-1- 1	1.3–61.8	-200–90	0.000099–0.142
	90%	168–390	-183–89	-1- 1	1.8–42.4	-183–89	0.000160–0.052
**Southeast**		***Bathymetry (m)***	***Slope***	***Northerness***	***Fine-scale BPI***	***VRM***	
	38%	32–597	0.0–86.4	-1- 1	-104–242	-0.00000012–0.75	
	70%	240–441	15.8–86.4	-1- 1	-98–149	-0.00000012–0.52	
	80%	249–430	19.5–74.1	-1- 1	-55–66	-0.00000012–0.05	
	85%	253–428	19.5–62.9	-1- 1	-42–61	-0.00000012–0.04	
	90%	253–424	19.8–57.1	-1- 1	-29–47	0.00–0.03	

Ranges of the contributing terrain attributes in order of importance for the trialed binary threshold values in the west, northeast and southeast region of Puerto Rico.

**Table 5:** Region-specific mean values for the retained variables within suitable habitat.

**Region**	**Threshold**	**Model Prediction**					
**West**		***Slope***	***Bathymetry (m)***	***VRM***	***Fine-scale BPI***	***Profile Curvature***	
	32%	11.9 ± 7.9	340 ± 124	0.0027 ± 0.0046	3.5 ± 32.1	-0.015 ± 0.460	
	70%	17.9 ± 7.9	299 ± 95	0.0046 ± 0.0059	18.1 ± 34.5	-0.103 ± 0.610	
	80%	18.8 ± 7.2	311 ± 74	0.0047 ± 0.0059	23.5 ± 32.1	-0.214 ± 0.580	
	85%	18.5 ± 6.3	326 ± 48	0.0040 ± 0.0036	31.8 ± 23.3	-0.223 ± 0.450	
	90%	17.6 ± 4.4	338 ± 36	0.0025 ± 0.0011	37.3 ± 15.8	-0.103 ± 0.306	
**Northeast**		***Bathymetry (m)***	***Broad-scale BPI***	***Northerness***	***Slope***	***Fine-scale BPI***	***VRM***
	66%	323 ± 49	-35.5 ± 53.3	0.54 ± 0.47	12.3 ± 7.7	-35.5 ± 53.3	0.0016 ± 0.0037
	70%	327 ± 43	-39.6 ± 53.2	0.51 ± 0.48	12.4 ± 6.6	-39.6 ± 53.2	0.0015 ± 0.0031
	80%	331 ± 39	-46.7 ± 52.7	0.41 ± 0.50	13.8 ± 6.0	-46.7 ± 52.7	0.0019 ± 0.0031
	85%	333 ± 37	-47.7 ± 53.2	0.34 ± 0.52	14.9 ± 6.0	-47.7 ± 53.2	0.0022 ± 0.0034
	90%	334 ± 35	-45.4 ± 52.1	0.24 ± 0.51	16.0 ± 5.5	-45.4 ± 52.1	0.0026 ± 0.0033
**Southeast**	** **	***Bathymetry (m)***	***Slope***	***Northerness***	***Fine-scale BPI***	***VRM***	
	38%	304 ± 87	30.9 ± 12.4	-0.62 ± 0.57	-5.0 ± 18.3	0.0056 ± 0.0056	
	70%	323 ± 44	33.7 ± 9.8	-0.55 ± 0.60	-8.6 ± 17.5	0.0048 ± 0.0095	
	80%	323 ± 42	34.4 ± 8.0	-0.47 ± 0.66	-10.0 ± 13.8	0.0039 ± 0.0053	
	85%	327 ± 41	35.5 ± 6.5	-0.40 ± 0.7	-10.8 ± 12.4	0.0037 ± 0.0044	
	90%	334 ± 38	36.4 ± 4.4	-0.25 ± 0.8	-11.0 ± 11.2	0.0032 ± 0.0034	

Mean values of the contributing terrain attributes, in order of importance, for the trialed binary threshold values in the west, northeast, and southeast regions of Puerto Rico.

**Fig 6:**
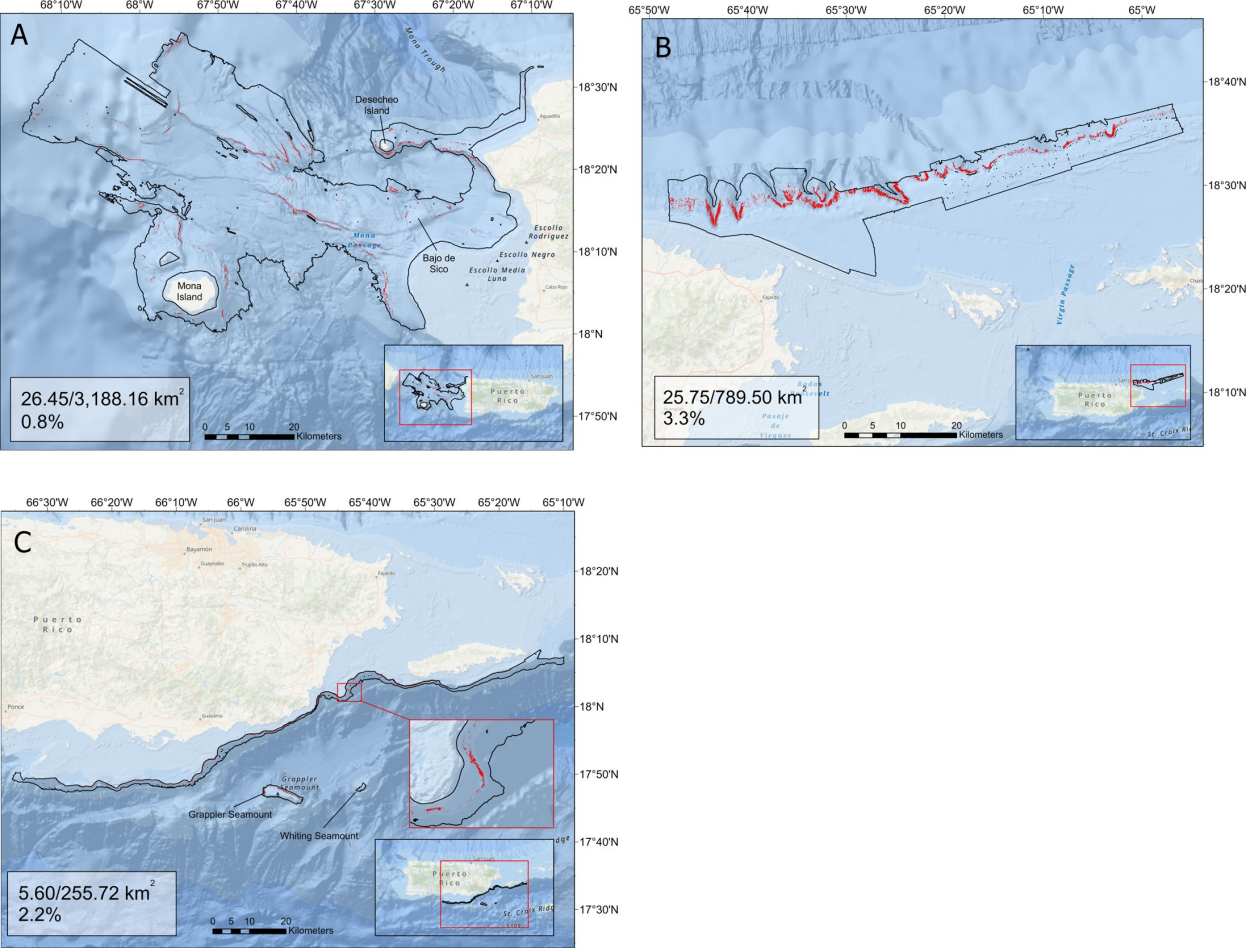
Region-specific binary habitat maps. Binary index of habitat suitability for queen snapper on the A) west, B) northeast, and C) southeast region of Puerto Rico. Total suitable habitat area (km^2^) out of total sampling frame area (km^2^), and percentage of total suitable habitat in lower left corner of each figure. The map layer used to generate this figure is from NOAA National Centers for Environmental Information and provided without restriction by the U.S. Government.

**Fig 7:**
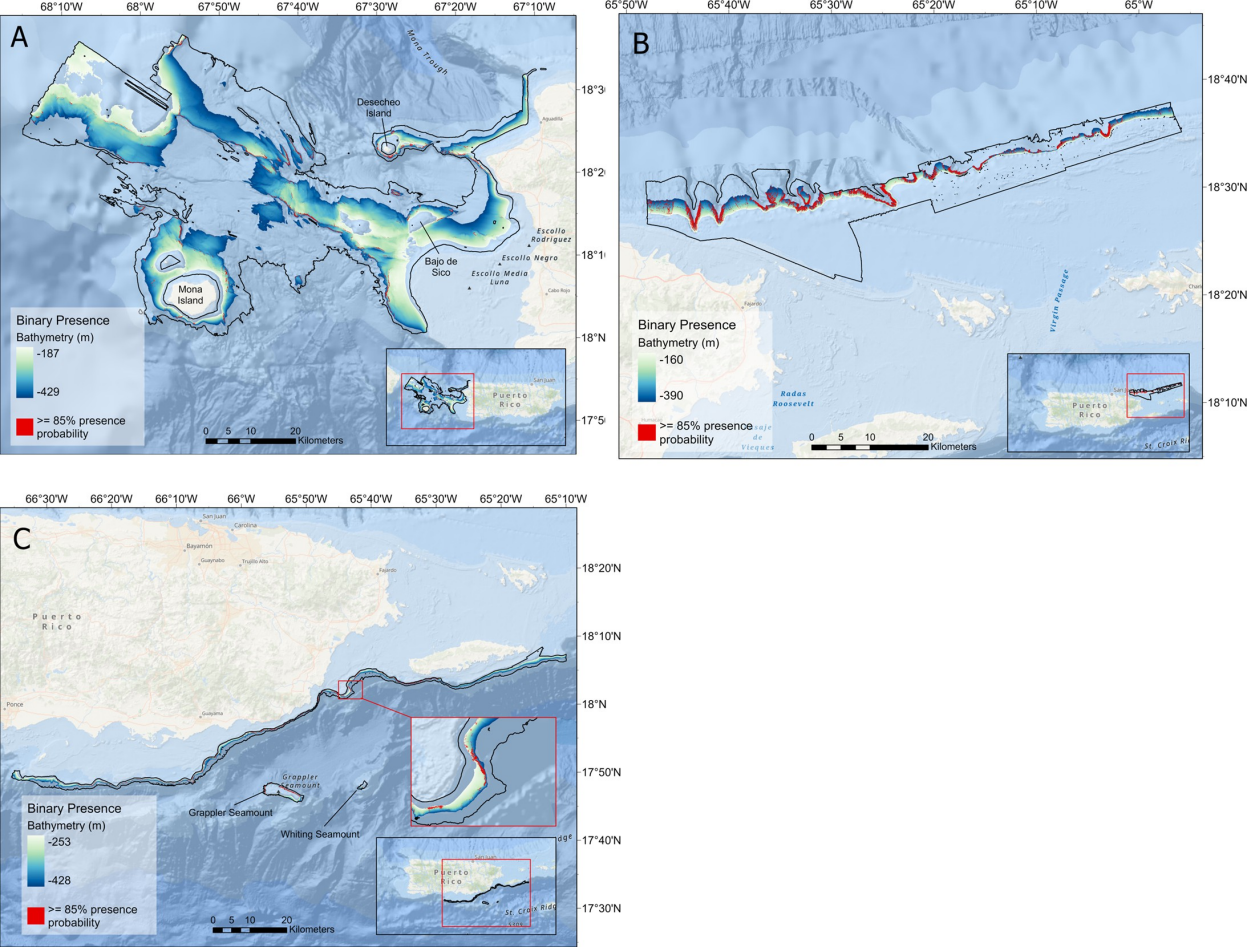
Region-specific depth restricted within suitable habitat. Total delimited sampling frame area bathymetry restricted to the 85% threshold model predicted depth range for the A) west, B) northeast, and C) southeast region of Puerto Rico (west: 187–429 m; northeast: 160–390 m; southeast: 253–428 m). The binary index of habitat suitability for queen snapper within 85% threshold model predicted depth range is depicted in red. The map layer used to generate this figure is from NOAA National Centers for Environmental Information and provided without restriction by the U.S. Government.

The percentage of suitable habitat using the 85% threshold was calculated for each region’s delimited (*i*.*e*., the 0–600 m depth range) multibeam mapping footprint. The western region contained the largest amount of suitable habitat with 26.45 km^2^ (0.8% of the delimited extent of the area), followed by the northeast with 25.75 km^2^ (3.3% of the delimited extent), and lastly the southeast with 5.63 km^2^ (2.3% of the delimited extent; Fig 6). Overall, when combined, we identified 57.77 km^2^ of suitable queen snapper habitat within the area covered by the delimited bathymetric data (1.4%). While these percentages of suitable habitat appear to be small, it is important to take into consideration that analysis examines the multibeam mapped data slightly beyond the known depth range for the species. As a comparison, the percentage of suitable habitat was calculated within the 85% threshold model predicted depth range (west: 187–429 m; northeast: 160–390 m; southeast: 253–428 m). This resulted in the largest amount of suitable habitat in the northeast with 19.0% of the extent, followed by 6.6% in the southeast, and 1.9% in the west (Fig 7); alternatively, the percentage of suitable habitat within the known depth range of queen snapper in literature (100–534 m) was 9.4%, 3.2% and 1.1% respectively.

### Variable contribution

One of the most influential variables in predicting queen snapper probability of presence across the three regions and both spatial resolutions was bathymetry, or depth (Table 6). The second and third most important variables differed between region and resolution. In the northeast and southeast bathymetry was the most important predictor of suitable habitat; however, in the west, slope contributed the greatest. Following bathymetry, the distribution in the northeast was driven by broad-scale BPI and northerness, and in the southeast slope and northerness. In the west, slope was followed by bathymetry and VRM.

**Table 6:** Region-specific variable importance.

Variable	West	Northeast	Southeast
**Bathymetry**	30.2	45.5	49.2
**Slope**	48.3	10.0	28.3
**Slope of Slope**	NR^a^	NR^a^	NR^a^
**Curvature**	NR^a^	NR^a^	NR^a^
**Plan Curvature**	NR^b^	NR^b^	NR^b^
**Profile Curvature**	4.2	NR^b^	NR^b^
**Vector Ruggedness Measurement (VRM)**	9.0	5.0	3.1
**Fine-scale BPI**	8.3	6.1	9.3
**Broad-scale BPI**	NR^a^	22.5	NR^a^
**Standard Deviation**	NR^a^	NR^a^	NR^a^
**Relative Deviation from Mean Value (RDMV)**	NR^a^	NR^a^	NR^a^
**Easterness**	NR^b^	NR^b^	NR^b^
**Northerness**	NR^b^	11.4	10.1

Environmental variable importance for each study region quantifying the relevance of any individual environmental variable that was used in ESDM. NR = not reported.

^a^Variables that were removed due to correlation with other variables (Spearman correlation coefficient > |0.65|).

^b^Variables that were removed due to variable relative importance < 3.0%.

### Habitat associations

Queen snapper were positively correlated with increasing depth with a mean depth ranging from 326–333 m, depending on region (Table 5). The depths at which probability occurrence peaks are 366 ± 7 m, 367 ± 4 m, and 323 ± 2 m in the west, northeast, and southeast regions, respectively (mean depth ± SD). Based on the variable relative importance, queen snapper presence was positively associated with areas of moderate to high slope, with low to moderate rugosity habitat (Tables 4 and 5). Queen snapper suitable habitat was modeled adjacent to features that are higher than the surrounding area (e.g., ridge-like features) and seamounts (Fig 7). The sign of the variables was seemingly affected by resolution, as fine-scale BPI was positive in the west, and negative in the northeast and southeast (Tables 4 and 5); additionally, broad-scale BPI was negative in the northeast. In the southeast, mean northerness was negative; whereas in the northeast, mean northerness was positive this indicates that north- and south-facing slopes may influence currents and thus habitat suitability for queen snapper. Mean profile curvature, which was found to contribute in the west, was negative although nearing zero.

### Uncertainty

**Fig 8:**
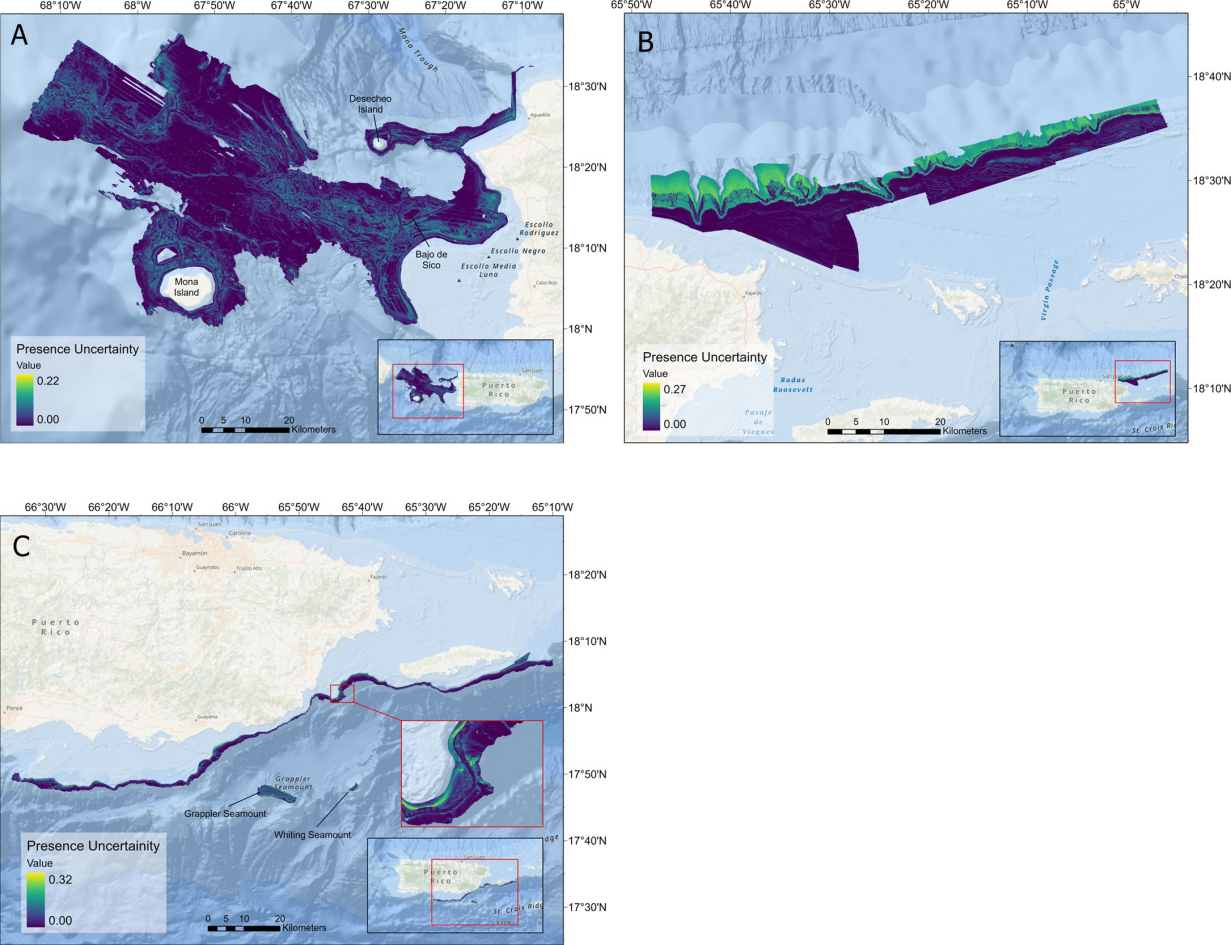
Region-specific probability of habitat suitability uncertainty maps. Uncertainty in ensemble projections of queen snapper occurrence in the A) west, B) northeast, and C) southeast region of Puerto Rico. The map layer used to generate this figure is from NOAA National Centers for Environmental Information and provided without restriction by the U.S. Government.

Uncertainty metrics were generated by the ESDM, which represent the between-model variance. In the three regions, areas with higher degrees of uncertainty were correlated with a moderate to low probability of suitable habitat (Fig 8). Overall, uncertainty remained fairly low in all regions with a maximum of 22% in the west, 27% in the northeast and 32% in the southeast, and means of 3%, 6%, and 5%, respectively.

## Discussion

### Habitat suitability and environmental drivers

The habitat suitability modeling conducted in this study is the first effort made to map queen snapper suitable habitat presence probability and associated uncertainty in the U.S. Caribbean. Areas of suitable habitat were predicted to occur throughout all three of the study regions. Taking into account the depth distribution of queen snapper and delimiting the multibeam data coverage across study regions the total area of suitable habitat increases to 3.6% when restricted to the 85% threshold model predicted depth range (Fig 6).

Bathymetry was one of the most consistent and significant variables in the ESDMs, with a relative model contribution between 30.2–49.2% (Table 6). The occurrence data collected for this project was limited to 80–500 m depths, which encompassed the known queen snapper depth distribution of 100–450 m at the time of sampling [17], whereas each sampling region included environmental variables from 0–600 m depths. Therefore, it is not surprising that bathymetry would be a driving factor in queen snapper habitat suitability models. There is a growing body of literature that uses depth and terrain attributes derived from it as a predictor of fish species and benthic species distribution [54, 55]. Moderate to high slope was an important variable in both the west and southeast ESDMs despite the difference in spatial resolution, with a relative model contribution of 48% and 28% respectively (Table 6). Although slope was not ranked as highly in the northeast ESDM, it did contribute 10%, highlighting a potential preference of queen snapper to areas of moderate slope. The differences in variable importance could be due to several factors including the difference in resolutions [56], or regional differences in the relationship between the direct and indirect variables.

With little biological data currently available for queen snapper, using surrogates to assist with determining management decisions is crucial [57]. Active acoustic data (*i*.*e*., bathymetry and backscatter and their derivatives) provide a proxy to better understand the distribution and complexity of marine benthic habitats and their relationship with direct and indirect surrogate variables. Although queen snapper habitat suitability and EFH have not been previously delineated, other species of Pacific *Etelis*, including *Etelis carbunculus* and *Etelis coruscans*, have been linked to specific benthic features through modeling. Potential indirect environmental drivers, specifically depth, have been shown to be the most important habitat predictor for the genus [58–60]. Our models indicate that queen snappers prefer a mean depth of 326, 333, and 327 m in the west, northeast and southeast, respectively, with 85% suitable habitat presence probability in ranges between 187–429 m, and 160–390 m, and 253–428 m, respectively. Misa *et al*., [58] also noted high-relief, hard-bottom areas as important habitat features for Pacific *Eteline* species. This is likely because of large benthic features, such as seamounts, pinnacles, and ridges retaining dense zooplankton populations due to upwelling from deeper depths, which in turn attracts larger predators such as the queen snapper [58, 61, 62]. When combined, terrain attributes can be linked to an environmental parameter such as food availability, which is difficult to calculate in situ. These observations of Pacific *Eteline* species are concurrent with the queen snapper habitat suitability maps we estimated for the Caribbean. The resulting models depict localized hot spots adjacent to Desecheo, Bajo de Sico, and Whiting and Grappler seamount (Fig 5), all areas of moderate to steep slope and rugosity often associated with harder substrates. Queen snappers were also predicted to be present in close proximity to elevated ridge features throughout the west and northeast coast. The negative mean fine-scale BPI in the northeast and southeast, and the negative mean broad-scale BPI indicates that at a higher resolution, queen snapper suitable habitat consists of areas that are lower than the surrounding area, such as depressions (fine-scale BPI) and valleys (broad-scale BPI). In contrast, queen snapper suitable habitat was correlated with positive fine-scale BPI and negative profile curvature in the west, although the layers were derived from 30 m resolution bathymetry as opposed to the 8 m resolution in the northeast and southeast.

The difference in variable relative importance highlights the idea that queen snapper could respond to habitat at multiple spatial resolutions, which has been shown in other species of marine fishes [63, 64]. An example of this for our case study with queen snapper can be seen with the slope variable. In the west, we measured slope at a 30 m resolution using a 3 x 3 window, overall characterizing slope over an area of 90 m by 90 m. In contrast, in the northeast and southeast we measured slope at 8 m using a 3 x 3 window, thus quantifying slope over 24 m x 24 m. The lower resolution data may be missing variations in slope that could be found at finer resolutions. Slope was found to be the highest contributing variable in the west (90 m) which may indicate that regional currents drive habitat suitability in those areas and not more localized currents that would be caught at higher resolutions. As this modeling approach was conducted in three distinct regions, it is possible and quite likely that the three regional models are showing differences in variable importance due to differences in the relationship between the direct (environmental variables such as temperature, salinity, food availability, etc.) and indirect surrogates (bathymetry, slope, rugosity, etc.) [54, 55, 57]. These relationships, while hard to identify and measure given the lack of data in this region and empirical linkage among variables, could potentially describe optimal food availability, refuge for juveniles, appropriate temperatures, favorable currents or salinity levels.

### Broader implications

The regional Fishery Management Councils are tasked with defining a species’ geographic range and habitat requirements by life stage. A system to analyze habitat information was developed under the Magnuson-Stevens Act Provisions (50 CFR Part 600) to better describe and identify EFH. This framework consists of four levels: 1) distribution data are available for some or all parts of the geographic range of the species; 2) habitat-related densities of the species are available; 3) growth reproduction or survival rates within habitats are available; and 4) production rates by habitat are available (50 CFR Part 600). Although queen snapper is included in the CFMC’s Reef Fish Fishery Management Plan, data on queen snapper habitat associations is scarce and the species does not have a well-defined EFH. The work and model-based maps highlighted in this study are critical to begin investigating level 1 by delineating the presence/absence of queen snapper and potential hotspots of occurrence for a portion of the species’ geographic range, in addition to investigating spatial scales (resolution and extent) and other contributing factors that influence species-environment relationships. Additionally, the predicted habitat suitability from queen snapper presence data does not take into consideration the species’ abundance, as such, it will be necessary to obtain data on the relative abundance to estimate potential yields.

### Future research

While this study is filling gaps in our knowledge of a deepwater snapper species, future considerations for modeling queen snapper and other deepwater snapper species in the U.S. Caribbean are important to note and could potentially bolster our understanding of habitat suitability. Presence-background ESDMs were utilized in this methodology, however the possibilities are nearly endless, and additional strategies could be tested to see if results outperform the strategy outlined in this study [65]. In addition, environmental data have been found to be lacking in several areas and the region could benefit from directed studies collecting high-resolution mapping and other benthic environmental data.

Backscatter data were available in areas that overlapped with our sampling universe; however, the data products were not standardized from the various sources. We attempted to utilize the methods outlined in Misiuk *et al*., [66] to harmonize data products in post-processing, but the backscatter files did not overlap to the degree needed to harmonize and create mosaics. Future work could focus on subdividing the regions into the extent covered by various backscatter datasets, integrating backscatter as a variable for evaluation in addition to the previously derived terrain attributes in this study.

While depth is a commonly used indirect surrogate for environmental variables such as water temperature, dissolved oxygen, and food abundance [57], our modeling approach could be improved upon by the addition of environmental variables such as water temperature, salinity, pH, and dissolved oxygen at depth. Due to the depths the target species occupies (> 100 m), temperate, salinity, and dissolved oxygen values that are consistent (from a period that overlaps with our sampling efforts), accurate (at depth recordings, not surface values), and spatially representative (within the spatial extent modeled) were not available for use. Additionally, surface-derived values for temperature and dissolved oxygen are not accurate representations of benthic depths due to limited mixing at depths deeper than 200 m. Future research should focus on collecting relevant environmental data for inclusion into habitat suitability modeling in the U.S. Caribbean.

Additionally, sediment observations from archived underwater videos could be integrated into a distribution model framework using a multiscale approach [*e*.*g*., 67]. The predicted sediment surfaces could then be incorporated as new variables in the queen snapper ESDM framework and further explored to improve predictions. Similar work could be conducted using deepwater coral species observations, as fishers around the island have noted entanglement with deepwater coral and sponges at common fishing grounds for queen snapper. Preliminary results from the fishery-independent data collected for use in this study suggest deepwater snapper, including queen snapper, were found to co-occur with deep-sea coral ecosystems. The survey recorded data at 471 survey sites, with 25% of sites documenting a minimum of one deep-sea coral or sponge species. A total of 17% of survey sites documented both deep-sea coral ecosystems and the presence of deepwater snappers [68].

Overall, the collection of high-resolution bathymetric and backscatter data and environmental parameters island-wide would allow for not only additional direct and indirect surrogates to the modeling approach, but would also enable us to combine separate regions into one large study region for comparison. As it stands currently, from an implementation standpoint combining the raster datasets from the three study regions where possible is too heavy to process with standard computers, as the mosaicking process would add large quantities of “no data” to each layer file where gaps in mapping exist. While the models may benefit slightly and predicted areas of habitat suitability would likely be more narrowly focused, the computational power needed is high and the process cumbersome. An additional concern with combining regional datasets is the loss of variability in our results caused by the specificity of each study area. As we do not understand all aspects that may drive queen snapper habitat suitability and the species presence in an area, the indirect nature of the variables in our approach may mean that the conditions driving the species’ habitat suitability in the three study regions are different. On an island platform such as Puerto Rico, there is a directionality when dealing with the data, specifically with regard to currents and food availability. Grouping the regions makes assumptions that the same variables drive the distribution of species across the island platform; specifically in the northeast and southeast which we know are likely different based on the geomorphology of the island platforms, dominant regional current directions, and the shallow shelf between the regions [69–72]. If queen snapper’s range was restricted to the deep ocean, assuming variable impacts are similar over a large extent is reasonable; however, with an island landmass such as Puerto Rico, we believe smaller regional models are important in addition to future comparison with a large mosaicked area to fully understand variable importance as data becomes available.

## Conclusions

This study used a state-of-the-art approach in the form of ensemble modeling to fill a gap in the literature regarding habitat suitability for a commercially and ecologically important deepwater snapper species. We took spatially-explicit seafloor variables derived from MBES bathymetry datasets and queen snapper presence datasets collected from fishery-independent methods to derive the probability of our target species inhabiting any particular area within our study regions. From modeling, we developed habitat suitability and uncertainty maps for each of the study regions. Our results demonstrate that seafloor characteristics function as effective predictors for queen snapper distribution across mesophotic and deepwater habitats. Our goal was to develop models and corresponding maps to be used as a tool to identify potential areas where queen snapper, a commercially and ecologically important species in the study regions, may reside when intensive field sampling may be cost-prohibitive. Additionally, our results highlight the potential effects of spatial variability in habitat suitability at multiple resolutions and the importance of considering this when modeling the presence probability of suitable habitat for a species. Based on this case study utilizing queen snapper, depth, and the orientation, arrangement and composition of benthic habitat features are key factors to integrate in spatial modeling and delineation of habitat suitability. While our results complement the limited knowledge that queen snapper can be found near oceanic islands and reefs on the continental shelf and upper slope [12, 58], they also serve to broaden our understanding of the spatial extent of queen snapper and highlight hotspots for potential management concerns such as EFH.

## Supporting information

S1 Table Algorithms used in species distribution modeling. Algorithms tested in each species distribution model, the dependent package used in R software, the default parameters utilized in each model, and the reference for each R package.
(DOCX)

S1 File Multibeam bathymetry files. National Centers for Coastal Ocean Science and United States Geological Society collected multibeam echosounder systems data.
(XLSX)
