# Supplementary material for: Mapping queen snapper (Etelis oculatus) suitable habitat in Puerto Rico using ensemble species distribution modeling
Source: PLoS One. 2024 Feb 26;19(2):e0298755. doi: 10.1371/journal.pone.0298755 (PMC10896535; doi:10.1371/journal.pone.0298755)
Supplement: S1 Table — Algorithms tested in each species distribution model, the dependent package used in R software, the default parameters utilized in each model, and the reference for each R package. (DOCX) [file pone.0298755.s001.docx]

| **Statistical method** | **Dependent package** | **Defaults** | **References** |
| --- | --- | --- | --- |
| MARS | earth | - Maximum degree of interaction (Friedman's mi) = 2 | Milborrow (2016) |
| MAXENT | dismo | - | Hijmans et al. (2016) |
| CTA | rpart | - Minimum number of observations in any terminal node = 1 - Number of cross-validations = 3 | Therneau et al. (2015) |
| GBM | gbm | - Total number of trees to fit = 2500 - Minimum number of observations in the trees terminal nodes = 1 - Number of cross-validations = 3 - Number of cross-validation folds to perform = 1e-03, if cv.folds > 1 then gbm, in addition to the usual fit, will perform a cross-validation | Ridgeway (2015) |
| ANN | nnet | - Maximum number of iterations = 500 | Venables and Ripley (2002) |
| RF | randomforest | - Number of trees = 2500 - Minimum size of terminal nodes = 1 | Liaw and Wiener (2002) |
| SVM | e1071 | - Epsilon parameter in the insensitive loss function = 1e-08. - If an integer value k>0 is specified, a k-fold cross-validation on the training data is performed to assess the quality of the model: the accuracy rate for classification and the Mean Squared Error for regression. By default, set to 3 | Meyer et al. (2015) |
